# Immunometabolism: Molecular Mechanisms, Diseases, and Therapies 2016

**DOI:** 10.1155/2017/8230298

**Published:** 2017-07-05

**Authors:** Jose C. Rosa Neto, Fabio S. Lira, Soumen Roy, William Festuccia

**Affiliations:** ^1^Immunometabolism Research Group, Institute of Biomedical Sciences, University of São Paulo (USP), 05508000 São Paulo, SP, Brazil; ^2^Exercise and Immunometabolism Research Group, Department of Physical Education, São Paulo State University (UNESP), Presidente Prudente, SP, Brazil; ^3^Cancer and Inflammation Program, Center for Cancer Research, National Cancer Institute, National Institutes of Health, Bethesda, MD 20892, USA; ^4^Department of Physiology and Biophysics, Institute of Biomedical Sciences, University of São Paulo, 05508000 São Paulo, SP, Brazil

In the second edition of the special issue titled “Immunometabolism: Molecular Mechanisms, Diseases, and Therapies,” a total of 37 manuscripts were received, and 16 of these were accepted. This issue demonstrated that nonresolving, chronic low-intensity inflammation not only is involved in the development and maintenance of several metabolic diseases such as visceral obesity, type 2 diabetes, dyslipidemias, atherosclerosis, hypertension, and cancer but also acts as an important linking factor between those conditions. Metabolic disease-associated inflammation is characterized by the recruitment of cells from both the innate and adaptive immune system to metabolic tissues followed by their polarization to a proinflammatory profile, resulting in the exacerbated local production of inflammatory mediators. These processes are mainly activated by the canonical Toll-like receptor- (TLR-) NF*κ*B signaling pathway, which can be triggered by several molecules such as the lipopolysaccharide from gut microbiota and saturated fatty acids, among others.

This special issue successfully attracted several interesting original and review articles addressing different aspects of the intricate relationships between metabolism and inflammation in health and disease. E. Nishida et al., for example, exploring the association between cardiovascular diseases and periodontitis, a chronic inflammatory disease that affects the periodontium, demonstrated that the acute-phase protein serum amyloid A, which is elevated in the liver and blood of apolipoprotein E-deficient, atherosclerosis-prone mice, promotes the expression of adhesion molecules in human aortic endothelial cells via TLR2, being therefore a candidate linking factor between periodontal disease and atherosclerosis. Subsequently, in an interesting, mechanistic study, Z.-Z. Guo et al. established a role for the c-Jun N-terminal kinase (JNK), a member of the mitogen-activated protein kinase (MAPK) family which is activated by inflammatory signals and other stress stimuli, as an important mediator involved in the formation of abdominal aortic aneurysm induced by angiotensin II plus nicotine, a major chemical component of cigarettes. These findings indicate that JNK inhibition may hold promise as a pharmacological target to attenuate smoking-induced abdominal aortic aneurysm formation. In an elegant study exploring the mechanisms underlying the immunomodulatory actions of bilirubin, K. F. Corral-Jara et al. found strong evidence indicating that, during hepatitis A virus infection, conjugated bilirubin differentially regulates CD4+ T lymphocytes and Tregs function by modulating intracellular pathways and by inducing changes in the proportion of Tregs expressing hepatitis A virus cellular receptor (HAVCR1/TIM-1). These findings may help in the elucidation of the mechanisms involved in hepatitis virus infection.

This special issue also gathered several studies that investigated the underlying mechanisms by which some nutrients and modulation of tissue oxygen levels may attenuate the chronic low-grade inflammation associated with metabolic diseases. Indeed, S. I. Pærregaard et al. investigated the involvement of membrane-free fatty acid receptor-4 (FFAR4), also known as GPR120, as mediator of the beneficial actions of *ω*3 fatty acids on metabolic health. By feeding *Ffar4* knockouts and heterozygous mice with either a control or an *ω*3-rich diet for 36 weeks, S. I. Pærregaard et al. show that FFAR4 signaling is not required for the anti-inflammatory and insulin-sensitizing effects mediated by *ω*3 fatty acids. G. Guan et al., on the other hand, presented interesting findings indicating that intake of a diet containing chitosan, polysaccharides found in insects, fungi, squid, oysters, krill, clams, and shellfish changes the composition of mice intestinal microflora by suppressing NF-*κ*B, TNF-*α*, and IL-6 and inducing a better control of inflammation and resolution of infection with *C. rodentium*. Moreover, V. Oliveira et al. showed that oligofructose supplementation in pregnancy and lactation induces the proinflammatory markers in dams and 90-day-old offspring and reduction in adiponectin pathway. In addition, F. Elbers et al. reported an impairment in the immunomodulatory and antimicrobial actions of the hepatic enzyme tryptophan 2,3-dioxygenase, which converts tryptophan, the essential amino acid for hosts and pathogens, to N-formylkynurenine, the precursor of the immune-relevant kynurenines, upon hypoxic conditions mimicking those occurring in vivo during infection and cancer, for example. These findings indicate that hypoxia might be detrimental for the appropriate host immune response towards relevant pathogens. In contrast to the deleterious effects of hypoxia, in an interesting study, S. Novak et al. reported that hyperbaric oxygenation treatment, which increases tissue oxygen content, oxidative metabolism, and production of reactive oxygen species (ROS), significantly reduces the severity of dextran sodium sulphate-induced colitis improving the inflammatory microenvironment in the gut mucosa, such an effect that seems to be mediated in part by the transcriptional factor hypoxic inducible factor 1 *α* (HIF-1*α*). Finally, regarding alternative treatments to inflammatory disease, W. Ren et al. reported that supplementation of drinking water of rodents with interferon tau, a type I interferon produced by trophectoderm cells of conceptuses of ruminant species, increased microbial diversity in the jejunum and ileum and decreased the expression of IL-17 in the intestines of normal and pathogen-infected mice, being therefore a candidate therapy strategy to treat the inflammatory intestinal diseases.

Two studies in this special issue were performed in humans aiming to further characterize obesity-associated inflammation. F.-I. Corona-Meraz et al., by evaluating the inflammatory and metabolic phenotype of subjects with obesity and insulin resistance, found that chemerin, an adipokine related to adiposity levels and fat distribution, is associated with obesity, dyslipidemia, and insulin resistance, whereas its receptor chemerin chemokine-like receptor 1 (CMKLR1) is associated only with obesity. C. Wang et al., on the other hand, by evaluating the gene expression profile of the visceral adipose tissue from lean and obese subjects from the Uyghur population, found a correlation between reduced expression of A2b adenosine receptor and the transcriptional factors Kruppel-like factors 4 and 5 with obesity-associated dyslipidemia and inflammation.

This special issue also brings some interesting review articles addressing several aspects of metabolic disease-associated inflammation. D. Ortuño-Sahagún et al., for example, reviewed recent evidence supporting the link between obesity and the pathogenesis of multiple sclerosis, a chronic autoimmune and inflammatory disease. P.-F. Bryan et al., on the other hand, reviewed the mechanisms involved in the regulation of cytokine profile by sphingolipids, the role of gut microbiota in providing signaling molecules that favor the communication between resident bacteria and intestinal cells, and the potential of sphingolipids and gut microbiota as targets or therapeutic agents for inflammatory bowel disease. H. J. Coelho Junior et al. reviewed the possible molecular mechanisms associated with muscle atrophy in stroke patients, as well as the modulatory effect of inflammation in this condition. G. van Niekerk et al. reviewed evidence suggesting that sickness-associated anorexia may be a mechanism by which autophagic flux is upregulated systemically and claim that some patients might benefit from permissive underfeeding. Finally, U. Nydegger et al. reviewed some approaches to sort out from big data the relevant results for patient care in precision medicine.

Therefore, in our opinion, this special issue brings new insights into the intricate mechanisms driving the inflammatory processes associated with metabolic diseases. We hope that these information will help to pave the way for the development of efficient strategies to prevent and treat these diseases.

*Jose C. Rosa Neto* *Jose C. Rosa Neto* *Fabio S. Lira* *Fabio S. Lira* *Soumen Roy* *Soumen Roy* *William Festuccia* *William Festuccia*

